# Current and future microbiological strategies to remove As and Cd from drinking water

**DOI:** 10.1111/1751-7915.12742

**Published:** 2017-07-11

**Authors:** James M. Byrne, Andreas Kappler

**Affiliations:** ^1^ Center for Applied Geoscience Geomicrobiology Sigwartstr. 10 Tuebingen 72076 Germany

## Introduction

Access to clean drinking water is one of the most fundamental of all human rights; however, many millions of people around the world, particularly in low‐income, developing countries, are regularly exposed to water sources that are contaminated by pathogenic bacteria, or toxic levels of pollutants such as the metalloid arsenic (As) and heavy metal cadmium (Cd) (Smedley *et al*., 2002; UN‐EP, 2010). These pollutants can lead to the development of many health issues such as rashes, lesions, cancers or even death (Smith *et al*., 2000; UN‐EP, 2010). Perhaps the most famous case of As release into drinking water supplies has affected South‐East Asia and the Bengal basin over recent decades. Widespread issues of pathogen‐contaminated surface water supplies and high mortality rate amongst under 5‐year olds led to the installation of millions of boreholes in the early 70s to access clean groundwater. Installing these boreholes led to changes in the local geochemistry and subsequent mobilization of As, which then became entrenched within the drinking water. Consequently, an approach conceived to ensure clean drinking water for millions of people, instead led the devastating effect of slowly poisoning them. Cadmium contamination is often associated with agricultural soils, due to the use of Cd‐containing phosphate fertilizers, mining or other industrial activities (Muehe *et al*., 2013a, b). These uses have led to contamination in groundwater via run‐off, in addition to the potentially harmful effects of bioaccumulation in plants, which can reach the food chain.

A sustainable approach to addressing these contamination issues is to either change the water source or treat the water source before it is consumed. Often, however, simply changing the water supply is not an option as entire regions may be affected and it becomes economically unviable to transport clean water over many hundreds of kilometres. Instead, it may be easiest and cheapest to treat the water before it is drunk or used for other purposes (e.g. agriculture, washing, etc.). Various remediation approaches have been used to date including membrane‐based technology, photochemical oxidation, ion exchangers, adsorbents, coagulants or flocculants (Berg *et al*., 2006; Hering *et al*., 2017). Here we discuss bioremediation methods which make use of microbiological processes in order to remove As and Cd from drinking water. Bioremediation strategies where bacteria are actively involved with the transformation of a species (e.g. through oxidation or reduction) are referred to it herewith as ‘direct microbial bioremediation’, whereas treatments by secondary processes (e.g. mineralization) stimulated by bacteria are referred to as ‘indirect microbial bioremediation’.

## Direct microbial bioremediation

Direct bioremediation approaches for As include those which directly reduce As(V) or oxidize As(III), which can be subsequently sorbed to or sequestered in minerals. As(III) oxidation can be mediated by heterotrophic bacteria, which use a detoxification mechanism to convert As(III) to As(V). Such strains include *Alcaligenes sp*. (Osborne *et al*., 1976). and *Variovorax sp*. MM‐1, which was noted to show the highest oxidation rates of all heterotrophs (1.23 × 10^−7^ μM^−1^ min^−1^ cell^−1^) (Bahar *et al*., 2013a). Autotrophic As(III)‐oxidizing bacteria use As(III) as an electron donor, coupled with O_2_ as electron acceptor and CO_2_ as carbon source. As(V) reduction has also been seen to take place as a detoxification strategy of bacteria via cytoplasmic arsenate‐reductase enzymes (Bahar *et al*., 2013b), although As(V) can also be used as a terminal electron acceptor for respiration, leading to As(III) remobilization, which is then removed by precipitation or complexation with sulfides (Bahar *et al*., 2013b). Such a sequestration strategy, however, would only be suitable in environments where sulfide and arsenic are present.

The direct microbial bioremediation of Cd remains poorly investigated. Cadmium predominantly exists in one oxidation state (+2) and can therefore not undergo the same kinds of redox reactions available to As. This means that any bioremediation must be achieved through bioaccumulation by Cd‐tolerant bacteria or plants. Recently, a Cd‐tolerant strain of *Geobacter sp*. Cd1 was identified (Muehe *et al*., 2013a); however, this is an iron(III)‐reducer and only mediates Cd removal via indirect microbial bioremediation rather than through any kind of metabolic pathway.

## Indirect microbial bioremediation

Indirect microbial bioremediation includes pathways, which do not enzymatically modify the As or Cd species, but instead stimulate other processes (e.g. iron oxidation) in which minerals are formed that subsequently sorb or sequester the heavy metals. Such mechanisms are evident in North Vietnam, where local people have developed household filters which simply contain sand. Iron‐ and arsenic‐containing contaminated groundwater is added at the top and then collected after filtration with an average of 80% As removed. The filters have relatively humble origins and were initially introduced to curb the unpleasant taste of the iron, which was characteristic of the local water supply (Berg *et al*., 2006) A rather serendipitous side‐effect was that as iron precipitated through abiotic and biotic iron oxidation, arsenic species bound to and were incorporated within the iron minerals (Berg *et al*., 2006; Voegelin *et al*., 2014). This effect was not only rapid, but highly effective with a common household drinking water filter able to supply enough water for a small household in just a few minutes (Nitzsche *et al*., 2015). Disadvantages of these filters include the fact that there are insufficient disposal methods available for the used filter material, meaning that As‐loaded iron rich sand is usually not properly disposed of and is simply discarded in the garden, street or river. Also, there is a risk that the presence of organic matter in the filter material could stimulate Fe(III)‐reducing bacteria, which can subsequently promote dissolution of the Fe minerals and release of As, especially after disposal of the used filter material (Islam *et al*., 2004). Another disadvantage of such filters includes the fact that they are only suitable when the ratio of Fe is high enough (Fe/As is ≥ 50) in the groundwater to form sufficient sorption site on fresh Fe mineral precipitates (Berg *et al*., 2006). Furthermore, whilst such an approach appears suitable for small‐scale applications, the ability to purify large volumes of water which could subsequently be used for agriculture remains out of reach.

Alternative strategies for heavy metal bioremediation consider the possible incorporation of As or Cd into different iron minerals such as magnetite. Magnetite is a magnetic, mixed‐valent iron mineral (containing both Fe^2+^ and Fe^3+^) which has been shown to incorporate both As(V) and As(III) produced by microbial Fe(III) reduction by *Geobacter sulfurreducens* (Islam *et al*., 2004; Coker *et al*., 2006). Other promising solutions focus on the sorption of As species onto iron oxides including magnetite nanoparticles (Mayo *et al*., 2007). The magnetic nature of the mineral means that it can be used to treat the contaminated water supply and either be removed my magnetic separation, or kept in place using a magnetic trap. Furthermore, the ability to control particle size of biogenic magnetite particles (Byrne *et al*., 2011) or selectively promote the formation of other biogenic iron minerals such as goethite (Dippon *et al*., 2015) means that biogenic approaches could be used to tailor the reactivity of biogenic iron minerals to selected bioremediation applications.

The problem associated with aforementioned studies, however, is that they were predominantly done *ex situ* and can thus be only done following extraction of the water from the aquifer. Sustainable strategies should perhaps instead focus on trying to promote the safe remediation of drinking water supplies *in situ*. A recent study based on a complex soil microcosm has shown that Cd can be incorporated into magnetite during microbial Fe(III) reduction by the addition of organic carbon (e.g. acetate or lactate) and promotion of anoxic and reducing conditions (by increasing water filled pore space) (Muehe *et al*., 2013b). In that study, the Cd was also shown to precipitate into carbonates (otavite, CdCO_3_) although the long‐term stability of such minerals was questioned as changes in pH could lead to the rapid remobilization of Cd back into the water supply. Nevertheless, we envision that a large‐scale bioremediation strategy could include the addition of organics into a large area and promotion of anoxic conditions through flooding. Finally, another approach could instead focus on stimulating sulfate‐reducing bacteria (e.g. *Desulfovibrio desulfuricans* or *Rhodobacter sphaeroides*) through the addition of FeSO_4_. The bacteria reduce sulfate to sulfide ions which then combine with heavy metals in the water and immediately precipitate, thus removing the heavy metal from the water (Bai *et al*., 2008; Joo *et al*., 2015). Cadmium sulfide is extremely stable and poorly soluble meaning that its mobility and bioavailability, and thus, its bioaccumulation could be lowered.

## Conclusion and future perspectives

Despite much research into the use of microbiology to treat metal pollutants such as As and Cd, it remains to be seen whether a sustainable, bioremediation strategy is achievable in the near future. Perhaps some of the best strategies will be to simply stimulate subsurface bacteria which can either directly change the oxidation state of the metal or incorporate it into minerals formed via alternative mechanisms. Alternatively, it might also be possible to treat the water supply after collection from an aquifer, but before consumption. Such a method will require a form of filtration, or precipitation, or the use of an adsorbent to immobilize the metal pollutant. The use of commercial adsorbent materials based on iron (oxyhydr)oxides is already used around the world to remove As from water supplies (Hering *et al*., 2017). Whilst such techniques mainly include abiotic processes, the role of microbes is still highly relevant, especially as it has been shown that bacteria can decrease the effectiveness of adsorbents designed to remove the heavy metal (Kleinert *et al*., 2011).

From a sustainable perspective, treating individual sources of water relies upon the social and economic status of the end user, with cultural customs sometimes preventing adequate maintenance or regular monitoring of the filters. Therefore, in future, larger‐scale operations are more likely to be able to meet the requirements for the millions of people who are affected by contaminated water worldwide. Some large‐scale operations have been tested, with one particular field‐based study set up in India in 2006 to treat As. In this field site, *Microbacterium lacticum* are used to oxidize As, which is then sorbed to activated alumina or charcoal (Mokashi *et al*., 2002). Such field sites could prove to one day be the best solution for low‐income communities.

## Conflict of interest

None declared.
